# Effect of Integrated Capacity-Building Interventions on Malaria Case Management by Health Professionals in Uganda: A Mixed Design Study with Pre/Post and Cluster Randomized Trial Components

**DOI:** 10.1371/journal.pone.0084945

**Published:** 2014-01-08

**Authors:** Martin Kayitale Mbonye, Sarah M. Burnett, Aldomoro Burua, Robert Colebunders, Ian Crozier, Stephen N. Kinoti, Allan Ronald, Sarah Naikoba, Timothy Rubashembusya, Jean-Pierre Van geertruyden, Kelly S. Willis, Marcia R. Weaver

**Affiliations:** 1 Training Department, Infectious Diseases Institute, Makerere University College of Health Sciences, Kampala, Uganda; 2 Accordia Global Health Foundation, Washington DC, United States of America; 3 Management Sciences for Health, Kampala, Uganda; 4 Department of Epidemiology and Social Medicine, Faculty of Medicine and Health Sciences, University of Antwerp, Antwerp, Belgium; 5 Department of Clinical Sciences, Institute of Tropical Medicine, Antwerp, Belgium; 6 Center for Human Services, University Research Co., LLC, Bethesda, Maryland, United States of America; 7 Fio Corporation, Toronto, Ontario, Canada; 8 Department of Medicine, University of Manitoba, Winnipeg, Manitoba, Canada; 9 Departments of Global Health and Health Services, University of Washington, Seattle, Washington, United States of America; The George Washington University Medical Center, United States of America

## Abstract

**Background:**

The Integrated Infectious Diseases Capacity Building Evaluation (IDCAP) designed two interventions: Integrated Management of Infectious Disease (IMID) training program and On-Site Support (OSS). We evaluated their effects on 23 facility performance indicators, including malaria case management.

**Methodology:**

IMID, a three-week training with two follow-up booster courses, was for two mid- level practitioners, primarily clinical officers and registered nurses, from 36 primary care facilities. OSS was two days of training and continuous quality improvement activities for nine months at 18 facilities, to which all health workers were invited to participate. Facilities were randomized as clusters 1∶1 to parallel OSS “arm A” or control “arm B”. Outpatient data on four malaria case management indicators were collected for 14 months. Analysis compared changes before and during the interventions within arms (relative risk = RR). The effect of OSS was measured with the difference in changes across arms (ratio of RR = RRR).

**Findings:**

The proportion of patients with suspected malaria for whom a diagnostic test result for malaria was recorded decreased in arm B (adjusted RR (aRR) = 0.97; 99%CI: 0.82,1.14) during IMID, but increased 25% in arm A (aRR = 1.25; 99%CI:0.94, 1.65) during IMID and OSS relative to baseline; (aRRR = 1.28; 99%CI:0.93, 1.78). The estimated proportion of patients that received an appropriate antimalarial among those prescribed any antimalarial increased in arm B (aRR = 1.09; 99%CI: 0.87, 1.36) and arm A (aRR = 1.50; 99%CI: 1.04, 2.17); (aRRR = 1.38; 99%CI: 0.89, 2.13). The proportion of patients with a negative diagnostic test result for malaria prescribed an antimalarial decreased in arm B (aRR = 0.96; 99%CI: 0.84, 1.10) and arm A (aRR = 0.67; 99%CI: 0.46, 0.97); (aRRR = 0.70; 99%CI: 0.48, 1.00). The proportion of patients with a positive diagnostic test result for malaria prescribed an antibiotic did not change significantly in either arm.

**Interpretation:**

The combination of IMID and OSS was associated with statistically significant improvements in malaria case management.

## Introduction

In 2010, the World Health Organization (WHO) estimated that, globally, 219 million people had malaria and between 490 and 836 thousand died due to malaria [1]. In the same year, WHO estimated that in Uganda between 5 and 14 million malaria episodes and between 13,288 and 25,723 deaths due to malaria occurred [1]. Malaria remains the major cause of morbidity and one of the leading causes of mortality in Uganda [2]. Malaria also accounted for up to 50 percent of outpatient visits at health facilities, 20 percent of all hospital admissions and over 20 percent of all hospital deaths [3]–[5].

Current WHO guidelines call for parasitological diagnosis for malaria and Artemisinin-based Combination Therapies (ACTs) for first line treatment of uncomplicated malaria [6]. Within Africa however, presumptive treatment remains common practice [7] as staff and supplies for good quality diagnosis remain in short supply [8]. In some instances, health workers even ignore parasitological diagnosis, and prescribe (often inappropriate) malaria treatment for patients with a negative diagnostic test result for malaria [7]–[10]. Clinical diagnosis has its limitations and can lead to misdiagnosis of malaria and result in mismanagement of non-malaria febrile illnesses, wastage of antimalarial drugs and subject patients to the potential risk of developing resistance [6], [11].

Recently, the Joint Uganda Malaria Program (JUMP) and Uganda Malaria Surveillance Program (UMSP) evaluated the effects of an integrated team-based malaria training and surveillance program on facility level performance in eight sites. In 2006–7, integrated team-based malaria training and surveillance significantly increased referral for parasitological diagnosis of malaria among patients with suspected malaria, and decreased prescription of antimalarial treatment for patients with a negative diagnostic test result for malaria [9], [12]. These interventions however, did not improve the percentage of patients with a positive diagnostic test result for malaria prescribed an antimalarial treatment or the percentage of patients with suspected malaria prescribed an appropriate antimalarial.

The Integrated Infectious Diseases Capacity Building Evaluation (IDCAP) sought to build on the JUMP results with two capacity-building interventions that had a wider scope. The interventions were: 1) the Integrated Management of Infectious Disease (IMID) training program for mid-level practitioners (MLP) and 2) on-site support (OSS). Their scope was malaria, pneumonia, tuberculosis, HIV and related infectious diseases. IMID consisted of courses and distance learning, while OSS was an educational outreach and Continuous Quality Improvement (CQI) package. The interventions reflected the latest understanding of how clinicians build both routine and complex reasoning skills as described in Miceli et al. [13].

We evaluated the effects of the interventions on 23 facility performance measures at 36 health facilities. Results of additional measures have also been reported elsewhere [14]. Two MLP at each facility received IMID. Health facilities were randomized as clusters (1∶1) to parallel arms: 18 sites in arm A received OSS in Time 1 from April 2010 to December 2010, and 18 sites in arm B served as a control. The combined effect of IMID and OSS was measured by the pre/post change in indicators in arm A between Time 0 (November 2009 to March 2010) and Time 1, and the effect of IMID was measured by the pre/post change in arm B. The cluster randomized trial component measured the additional effect of OSS as the difference in the pre/post change across arms. The facilities were randomized as clusters, because the JUMP evaluation showed that the performance indicators depended on a team of clinicians, laboratory professionals and data entry staff rather than individuals.

The protocol for the interventions and evaluation is described in Naikoba et al. [15]. The full protocol and supporting CONSORT checklist are available as supporting information; see Checklist S1 and Protocol S1. The primary objective of this article was to report the detailed analysis of the effect of IMID and OSS on four facility performance indicators for malaria case management. The secondary objective was to conduct exploratory analyses of alternative performance indicators for malaria diagnoses.

## Methods

### Participants

The 36 sites were health center IVs (HCIV) or comparable facilities drawn from all four administrative regions of Uganda: central, east, north and west. The district health system of Uganda is organized by health subdistrict, which is usually led by a HCIV. Each HCIV provides basic preventive, and curative care to a population of 100,000 people, as well as referral services, for the health subdistrict [2]. MLP are among the cadre listed on the staffing norms for a HCIV, as well as medical officers, pharmacists, nursing assistants, other allied health professionals, administrative staff, support staff, and other staff. Two MLP from each site participated in IMID. The facility and MLP inclusion criteria were described in Miceli et al. and Naikoba et al. [13], [15]. Briefly, two key inclusion criteria for facilities were a functioning laboratory and accreditation to prescribe anti-retroviral therapy. Two key inclusion criteria for MLP were cadre (clinical officer, registered nurse, or registered midwife) and devoting the majority of their time to clinical care. All facility staff were invited to participate in OSS and all outpatients participated during the normal process of receiving care.

### Interventions

#### IMID training program

The IMID training program began with a three-week core course at the Infectious Diseases Institute (IDI) in Kampala, followed by two, one-week boost courses at 12 and 24 weeks after the core course, and distance learning as described in Miceli et al. and Naikoba et al. [13], [15]. Building on the WHO's integrated approach to training, such as the Integrated Management of Child Illnesses (IMCI) and Integrated Management of Adult Illnesses (IMAI) curricula, IDCAP developed a training program for malaria, tuberculosis, HIV and related infectious diseases for children, adults and pregnant women.

The IMID curriculum was case-based, and fever and malaria case management were the focus of six of 39 sessions [13]. The six sessions on malaria are displayed in Table 1. Sessions 5 and 6 were based on the JUMP curriculum, which was in turn based on guidelines issued by the Uganda Ministry of Health, Malaria Control Program [16], and the World Health Organization [6], including IMCI. Subsequent sessions introduced cases of increasing complexity. Malaria was also discussed in several other sessions with reference to differential diagnoses. Two clinical decision-making guides (CDG) on fever case management and malaria case management summarized the key decision-making steps to consider when managing patients with fever. The IDCAP training materials can be requested at: http://www.accordiafoundation.org/IDCAP/innovations-in-training, including CDGs and a distance-learning version with audio lectures.

**Table 1:** Malaria case management sessions in the IMID curriculum.

Session	Session Focus	Session details
Session 5	A patient with fever	Diagnosis and management of uncomplicated malaria
Session 6	A patient with fever	Diagnosis and management of non-malarial causes of fever
Session 23	A child with fever	Diagnosis and management of fever in children
Session 24	A sick neonate	Diagnosis and management of fever/hypothermia in the young infant
Session 36	A patient with persistent fever	Diagnosis and management of persistent fever (focus HIV-infected)
Session 38	A patient with fever	Diagnosis and management of complicated malaria

#### OSS sessions

The OSS sessions were delivered for two days every month for nine consecutive months by four-person mobile teams each consisting of a medical officer with expertise in continuous quality improvement (CQI), a clinical officer, a laboratory technologist and a registered nurse. Each monthly OSS session was devoted to a specific topic and all OSS topics are reported in Miceli et al, and Naikoba et al. [13], [15]. The second monthly topic was fever case management. During day one of the OSS session on fever case management, a multi-disciplinary team (MDT) session for all the health cadres at the facility sought to empower health workers with knowledge and skills to assess a febrile patient, properly diagnose malaria, and treat uncomplicated malaria. Three breakout sessions were organized: 1) a session for clinical officers and registered nurses on clinical management of complicated malaria, 2) a session for enrolled nurses and midwives on malaria in pregnancy, and 3) a laboratory staff session on laboratory testing for malaria. Then, individual mentoring sessions with selected clinicians and laboratory professionals reinforced key competencies required for proper management of malaria. Day two was devoted to CQI activities, as well as additional mentoring sessions. The two CQI activities were a meeting of facility CQI teams to review data on facility performance indicators, and an MDT session on patient flow and processes of care for patients with fever, in order to identify problems and implement strategies to address them.

As a training and CQI program, the medical interventions were not at the discretion of the investigators. For CQI, the facilities were assigned to a set of goals that were associated with performance indicators, but not to medical interventions. The facilities chose six of 13 goals and then created or adopted the processes of care to attain them. CQI is based on the philosophy that facility teams are more motivated when they select the goals, and facility teams create or adopt more effective processes, because they are most familiar with their work environment. The two goals for malaria case management were: 1) All patients with suspected malaria to have results for blood smear or rapid diagnostic test, and 2) To reduce the proportion of patients with a negative diagnostic test result for malaria treated with antimalarials. Fifteen and 12 of 18 sites in arm A chose to focus on goals 1 and 2 respectively. This article reports the analysis of all facilities in arm A. Weaver et al. (unpublished manuscript) conducted a sensitivity analysis with the arm A sites that chose to focus on the malaria goals and the results were similar to the ones reported in this article.

### Outcomes

**Figure 1:**
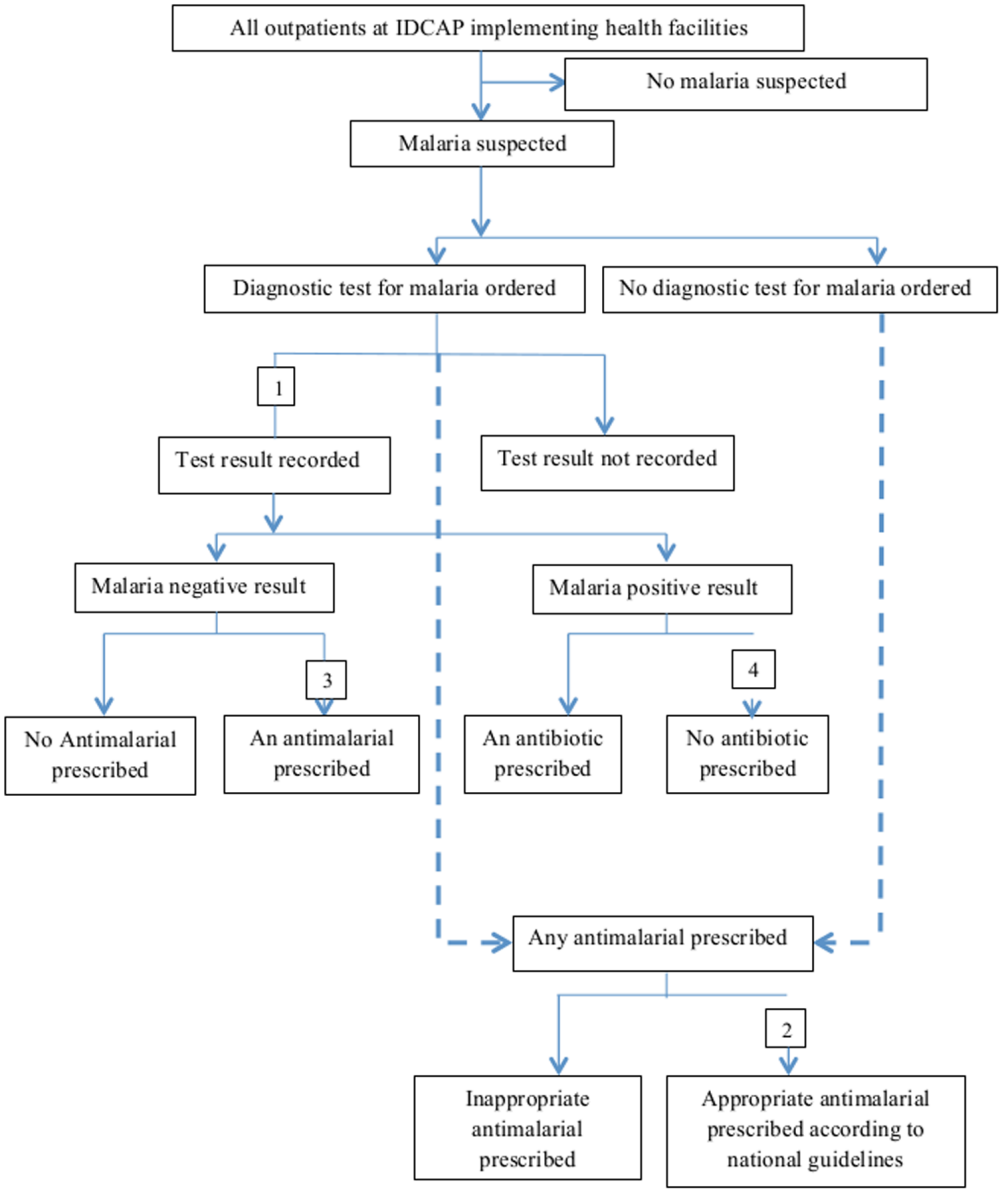
Malaria Case Management Analysis framework.

The four facility performance indicators for malaria case management are presented in Figure 1 and are defined in Table 2. The three alternative indicators for malaria diagnosis are defined in Table 2.

**Table 2:** Indicators for malaria case management.

	Indicator name	Indicator definition
	Facility performance indicators for malaria case management	
1	Proportion of patients with suspected malaria for whom a diagnostic test result for malaria was recorded.	**Numerator**: Number of patients with suspected malaria for whom a diagnostic test result for malaria, either microscopy or rapid diagnostic test, was recorded.
		**Denominator**: Total number of patients with suspected malaria.
2	Estimated proportion of patients who received an appropriate antimalarial	**Numerator**: Estimated number of patients who received an appropriate antimalarial.
		**Denominator**: Total number of patients prescribed any antimalarial.
3	Proportion of patients with a negative diagnostic test result for malaria prescribed an antimalarial	**Numerator**: Number of patients with a negative diagnostic test result for malaria who were prescribed an antimalarial.
		**Denominator**: Total number of patients with a negative diagnostic test result for malaria.
4	Proportion of patients with a positive diagnostic test result for malaria prescribed an antibiotic	**Numerator**: Number of patients with a positive diagnostic test result for malaria who were prescribed an antibiotic.
		**Denominator:** Total number of patients with a positive diagnostic test result for malaria.
	**Alternative indicators for malaria diagnosis**	
1	Proportion of patients with a malaria diagnosis among those with a negative diagnostic test result for malaria	**Numerator**: Number of patients with a negative diagnostic test result for malaria with a malaria diagnosis.
		**Denominator**: Number of patients with a negative diagnostic test result for malaria.
2	Proportion of patients with a malaria diagnosis among those with a positive diagnostic test result for malaria	**Numerator**: Number of patients with a positive diagnostic test results for malaria with a malaria diagnosis.
		**Denominator:** Number of patients with a positive diagnostic test result for malaria.
3	Proportion of patients with a positive diagnostic test result for malaria among those with a malaria diagnosis	**Numerator**: Number of patients diagnosed with malaria with a positive diagnostic test result.
		**Denominator:** Total number of patients with malaria diagnosis.

Information about drug availability was missing for some patients and as a result, the number of patients who received an appropriate antimalarial treatment was estimated from two intermediate measures: (a) proportion of patients prescribed an appropriate antimalarial among those with any antimalarial prescription, and (b) proportion of patients that received an appropriate antimalarial among those with an appropriate prescription and data about drug availability. The number of patients who received an appropriate antimalarial was estimated for each facility month as the product of the number of patients prescribed an appropriate antimalarial and (b).

### Data Sources and Variable Definitions

Data were collected using a Uganda Ministry of Health Medical Form 5 (MF5), initially modified by UMSP [9] to link the clinical data to laboratory data, as well as to include tick boxes for history, laboratory investigations, diagnoses and drug prescriptions. It was further revised by IDCAP to capture detailed information on drug availability among other things. The form was reported in Mbonye et al. (unpublished manuscript). The data relevant for malaria case management included: fever or history of fever in the history section; blood smear for malaria, parasite density, and rapid diagnostic test for malaria in the laboratory section; malaria diagnosis (during and not during pregnancy) in the diagnosis section; and the treatment data described below in the treatment section.

Patients with suspected malaria were defined as all patients with a fever, referred for malaria laboratory testing, or given a clinical diagnosis of malaria as evidenced by either a record of malaria diagnosis or an antimalarial prescription.

An appropriate antimalarial referred to quinine or artesunate and the following ACTs: artemether & lumenfantrine, artesunate & amodiaquine, or dihydroartemisinin & piperaquine phosphate (Duocotecxin®).

Any antimalarial treatment included the appropriate antimalarials listed above and three drugs that did not comply with Uganda national guidelines: amodiaquine alone, chloroquine, and sulfadoxine/pyrimethamine (SP) (Fansidar®).

Any antibiotic treatment referred to 12 drugs listed on the MF5: Amoxicillin, Benzyl Penicillin, Chloramphenicol, Ciprofloxacin, Cloxacillin, Cotrimoxazole, Doxycycline, Erythromycin, Gentamicin, Metronidazole, PPF/Procaine Penicillin, Tetracycline. Data on these drugs was elicited by checking boxes on the MF5. It also included 19 antibiotics recorded as “other drugs:” Ampiclox® (Ampicilllin & Cloxacillin), Ampicillin, Ampicillin & Gentamicin, Azithromycin, Cefalexin, Cefixime, Ceftriaxone, Cefuroxime, Co-amoxiclav, Dapsone, Dicloxacillin, Gatifloxacin, Levofloxacin, Nalidixic acid, Nitrofurantoin, Ofloxacin, Pencillin (generic), Perfloxacin, Phenoxymethyl Penicillin.

### Data Collection

Individual data were collected on every outpatient from November 2009 to December 2010. The MF5 was completed by various people involved in the process of care including but not limited to: records and clinical staff at the patient reception desks, clinicians during history taking, diagnosis, prescription and/or referrals, laboratory professionals during laboratory investigations and pharmacists/dispensers when dispensing prescribed drugs. Completed MF5 were electronically captured using Epi Info Version 3.2™ (U.S. Centers for Disease Control and Prevention, Atlanta, GA). Beginning in March 2010, data were entered by a Data Entry Assistant (DEA) stationed at each site and then electronically transmitted by an internet modem or a smartphone to IDI for further cleaning and analysis. The data from each site were merged using Microsoft Excel® (Microsoft Corporation, Redmond, WA, USA) and after merging exported to Stata® version 11 (Stata Corp, College station, Texas, USA) for analysis. Using systematic random sampling, 5% of the completed MF5 were selected and re-entered carefully by a data technical support team from IDI and compared with ones entered by the DEA using the Epi Info “data compare” command. Results indicated over 99% level of concordance.

### Randomization

The 36 sites were randomized as clusters (1∶1) to parallel arms: 18 sites in arm A (OSS in Time 1 from April 2010 to December 2010) and 18 sites in arm B (served as control during Time 1). The sites were randomized in two strata to balance allocation of two other on-site interventions across arms: a) previous participation in a national CQI program for HIV prevention and care and b) previous or current participation in the Baylor International Peadiatric AIDS Initiative (See http://www.bipai.org/Uganda/ for more information). The effects of these on-site interventions could have been confounded with OSS, which was also an on-site intervention. Site identification and selection was done by the Principal Investigator (MRW) and program managers. Randomization was conducted by the IDCAP biostatistician on 23^rd^ February 2010. It was not possible to conceal site allocation to project staff and participating health professionals during the intervention.

### Ethical Considerations

IDCAP was reviewed and approved by the School of Medicine Research and Ethics Committee of Makerere University (reference number 2009-175) and the Uganda National Council of Science and Technology (reference number HS-722). The IDCAP proposals were to electronically capture and extract data from the Ministry of Health, Health Management Information System (HMIS) for evaluating IDCAP interventions on facility performance indicators, a process which began after their approvals. IDCAP reinforced the HMIS, which routinely collects patient data at the facilities, a process that was ongoing before the committees' approvals and after IDCAP ended. Data collected for the HMIS was part of routine surveillance at the health facilities and did not require ethical approval. Informed consent of participants was not required, because the interventions were evaluated on facility performance using HMIS forms and registers rather than individual performance. Informed consent of patients was waived for the indicators reported in this article. The University of Washington Human Subjects Division determined that IDCAP did not meet the regulatory definition of research under 45 CFR 46.102(d).

### Sample Size

Sample size calculations were based on testing the effect of OSS on facility performance with the facility as the unit of analysis rather than the patient as described in Naikoba et al. [15]. The patient data were anonymous, and we couldn't control for multiple visits by the same patient. Estimates based on patient as the unit of analysis would have underestimated the standard errors. We used the facility as the unit of analysis where the observation was a percentage, such as the percentage of patients with suspected malaria referred for parasitological diagnosis. It is rare to have indicator data on multiple facilities and we were fortunate to have the JUMP data on the average percentage and standard error across facilities for the sample size calculations [12].

Briefly, the calculations were based on JUMP results showing roughly a 20% mean absolute improvement in two malaria indicators: 1) percentage of patients with suspected malaria referred for parasitological diagnosis of malaria, and 2) percentage of patients with a negative diagnostic test result prescribed antimalarial treatment [16]. To detect a 20% mean absolute difference between the intervention and control arm with a power of 80% and an alpha of 0.05, 18 sites were needed in each arm. The calculations assumed a Gaussian distribution of the indicators and were performed with Stata version 10.

### Statistical Methods

Descriptive statistics were used to describe patient populations across arms, time, and age groups. The effects of IMID and OSS were analyzed as binomial experiments with facility-month as the unit of analysis. For example, the number of patients with suspected malaria for whom a diagnostic test result for malaria was recorded would be the numerator or number of “successes” and the number of patients with suspected malaria would be the denominator or number of “trials”. We used a generalized linear model for the proportion of patients managed appropriately for a given indicator with main effects for arm, time period and their interaction. To analyze the effects of the interventions on each indicator, the pre/post difference in arm B measured the effect of IMID, the pre/post difference in arm A measured the combined effect of IMID and OSS, and the incremental difference between arm A and B measured the effect of OSS. In contrast to analyzing indicators as a proportion, the binomial experiments allowed the precision of the estimates to vary across facilities with different numbers of patients. All regression analyses were clustered on the facility with robust standard errors to adjust for over-dispersion and using the Poisson instead of the binomial family and a log link to estimate the relative risks (RR) [17]. Results for the interventions were presented with 99% confidence intervals (99% CI). Tests were based on a 1% level of significance because there were multiple comparisons. All analyses were performed with Stata® version 11.

Independent variables considered in the analysis were; patient age, facility level, facility type, BIPAI supported, CQI experienced, DEA stationed at site, malaria endemicity, and three staffing variables. The covariates apply to the sample as a whole; we did not interact the covariates with the variable for the main effects. Each site was assigned to one of four categories for endemicity as reported in Adoke et al. [18]: 1) very low or no malaria (prevalence <5% in children), 2) low (prevalence 5–10% in children), 3) medium-high (prevalence 10 to 50% in children except during seasonal peaks, and 4) very high (prevalence greater than 50% in children). The staffing variables were measured in quartiles: 1) proportion of ideal clinical staff assigned to the facility at baseline, where ideal was defined by Uganda Ministry of Health staffing norms [2], 2) number of clinical staff who saw at least five patients during a month divided by the number of patients at the facility during that month, and 3) proportion of laboratory professionals assigned to the facility at baseline as per the staffing norms.

The pre/post time periods for both arms were not the same, because the MLP in arm A attended the first two IMID sessions (March 15^th^–April 2^nd^ 2011 and April 12^th^–30^th^) while those in arm B attended the last two sessions (May 3^rd^–21^st^, and June 7^th^–25^th^ 2011). Therefore, in arm A, baseline (Time 0) was five months from November 2009 to March 2010. In arm B it was seven months from November 2009 to May 2010. In arm A, the intervention period (Time 1) began in April 2010 and extended for nine months to December 2010. In arm B it began in June 2010 and extended for seven months to December 2010.

Several sensitivity analyses were performed including estimating variance with bootstrapping rather than robust standard errors. SP monotherapy is considered appropriate for Intermittent Preventive Treatment during pregnancy, so we conducted a sensitivity analysis that omitted women aged 15–49 treated with SP monotherapy and not diagnosed with malaria, who make up 4.67% (1,754) of all inappropriate diagnoses. In addition, estimates were repeated without variables such as DEA stationed at site and the staffing variables that could potentially have been collinear with the main effects. Plotting of residuals and Cooks distance regression diagnostics were performed and the main model estimates were repeated with outliers and influential observations respectively omitted.

## Results

### Participant Flow

**Figure 2:**
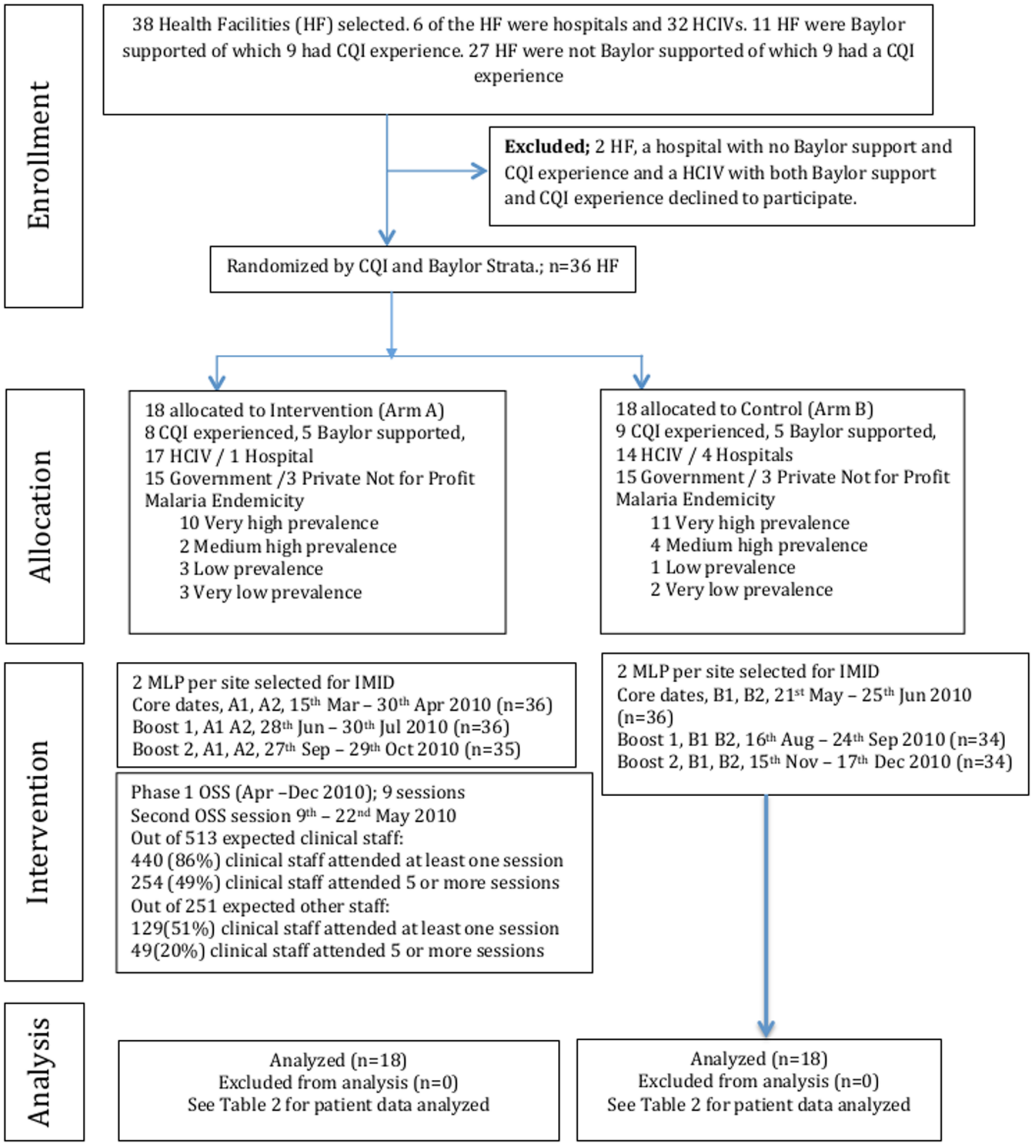
Consort Flow Diagram – Recruitment and Randomization.

A total of 36 out of 38 sites that met the inclusion criteria were enrolled in IDCAP as shown in Figure 2; 17 and 10 sites were in the CQI and BIPAI strata respectively. In the random allocation process, the sites were evenly distributed between arms by facility type (three private-not-for-profit sites in each arm) and malaria endemicity (10 sites with very high endemicity in arm A and 11 in arm B). Only one of the five hospitals however, was randomly assigned to arm A. Two MLP from each site participated in the IMID. A total of 45 (24 in arm A and 21 in arm B) clinical officers, 23 (12 in arm A and 11 in arm B) registered nurses and four (all in arm B) registered midwives participated in IMID. One MLP in arm A did not participate in the second boost course, and three MLP in arm B did not participate in at least one of the boost courses.

For arm A, during the second OSS session on fever case management, 276 (64%) out of 431 eligible clinical and laboratory staff attended the MDT, 107 (60.1%) out of 178 attended the mentorship session, 101 (35.8%) out of 282 attended cadre specific breakout sessions and 266 (61.7%) out of 431 attended the CQI session.

During the 14 months included in the analysis, data on 777,667 outpatients were collected and 753,074 were analyzed. Data on age were missing for 24,593 (3.3%) of outpatients who were omitted from the analysis.

### Recruitment

The sites were recruited between March and September 2009. Identification and recruitment of IMID participants took place between June 2009 and February 2010. Their registration and consent process took place between December 2009 and March 2010. Recruitment and registration of OSS participants began in April 2010 and continued during the intervention; all staff were encouraged to attend OSS sessions irrespective of previous attendance. Outpatients were seen when they sought care and their consent process was waived.

### Baseline

Table 3 summarizes the patient population by age and EIR. At Time 0, data were collected on 290,183 outpatients; the smaller proportion of these patients were in arm A (33%), largely because more hospitals were randomly assigned to arm B and Time 0 was two months longer in arm B. In arm A and arm B respectively, 28% and 31% of the patients were children under five years. The proportion of patients with suspected malaria was generally higher among children under five years (85% in arm A and 87% in arm B) than in older patients (61% in arm A and 58% in arm B). Seropositivity rate for diagnostic tests for malaria was generally higher in children under fives years than it was in older patients. Baseline results for each indicator are reported in Tables 4, 5, and 6.

**Table 3:** Outpatient population by age and entomological inoculation rate.

	Arm A		Arm B	
	(n = 18)		(n = 18)	
	Time 0	Time 1	Time 0	Time 1
Facility indicators	N (%)	N (%)	N (%)	N (%)
***Total patients***	**94,812**	**235,784**	**195,371**	**227, 107**
***By age***				
Under 5	26,264 (28%)	60,988 (26%)	59,921 (31%)	60,211 (27%)
5 and over	68,548 (72%)	174,796 (74%)	135,450 (69%)	166,896 (73%)
***By EIR***				
Very low	11,089 (12%)	27,071 (11%)	12,524 (6%)	15,010 (7%)
Low	20,935 (22%)	49,811 (21%)	8,950 (5%)	9,449 (4%)
Medium – High	7,526 (8%)	16,563 (7%)	44,364 (23%)	44,948 (20%)
Very High	55,262 (58%)	142,339 (60%)	129,533 (66%)	157,700 (69%)
***Patients with suspected malaria (% of total patients)***	**63,729 (67%)**	**144,397 (61%)**	**130,540 (67%)**	**146,912 (65%)**
***By age***				
Under 5	22,228 (85%)	49,779 (82%)	51,930 (87%)	51,116 (85%)
5 and over	41,501 (61%)	94,618 (54%)	78,618 (58%)	95,796 (57%)
***By EIR***				
Very low	4,768 (43%)	10,823 (40%)	4,165 (33%)	6,053 (40%)
Low	15,072 (72%)	32,051 (64%)	5,408 (60%)	4,339 (47%)
Medium – High	5,451 (72%)	9,965 (60%)	28,874 (65%)	27,370 (61%)
Very High	38,438 (70%)	91,558 (64%)	92,093 (71%)	109,090 (69%)
***Patients with positive test results for malaria (% of patients tested for malaria)***	**11,498 (49%)**	**31,996 (44%)**	**20,867 (46%)**	**20,293 (30%)**
***By age***				
Under 5	5,921 (64%)	15,510 (59%)	10,860 (56%)	10,434 (50%)
5 and over	5,577 (40%)	16,486 (35%)	10,007 (39%)	9,856 (31%)
***By EIR***				
Very low	410 (14%)	635 (8%)	252 (14%)	136 (7%)
Low	1,925 (45%)	4,257 (30%)	380 (26%)	62 (10%)
Medium – High	922 (61%)	2,428 (44%)	6,915 (44%)	4,879 (31%)
Very High	8,241 (57%)	24,675 (55%)	13,320 (51%)	15,215 (44%)

**Table 4:** Adjusted relative risk across time periods and arms for parasitological diagnosis of malaria.

	Proportion of patients with suspected malaria for whom a diagnostic test for malaria was ordered		Proportion of patients with suspected malaria for whom a diagnostic test for malaria was recorded	
Sub groups	Under 5	5 and above	Under 5	5 and above
**Part I: Percentage**				
**Arm A**				
Time 0	10,409 (47%)	15,572 (38%)	9,292 (42%)	13,947 (34%)
Time 1	30,281 (61%)	51,786 (55%)	26,413 (53%)	47,033 (50%)
**% Change**	**14%**	**17%**	**11%**	**16%**
**Arm B**				
Time 0	21,465 (41%)	28,905 (37%)	19,228 (37%)	25,980 (33%)
Time 1	23,526 (46%)	34,922 (36%)	20,972 (41%)	31,416 (33%)
**% Change**	**5%**	**1%**	**4%**	**0%**
**Part II: Regression**	**aRR (CI)**	**p-value**	**aRR (CI)**	**p-value**
**Arm A:** T1 – T0	1.28 (0.97, 1.68)	0.021	1.25 (0.94, 1.65)	0.045
**Arm B:** T1 – T0	0.99 (0.86, 1.14)	0.856	0.97 (0.82, 1.14)	0.636
**Arm A vs. Arm B:**				
T1-T0 **(aRRR)**	1.29 (0.94, 1.77)	0.036	1.28 (0.93, 1.78)	0.049
**Covariates**				
Age (1 = Under 5)	1.16* (1.03, 1.31)	0.002	1.14* (1.00, 1.30)	0.009
Facility level (1 = Hospital)	1.07 (0.63, 1.81)	0.736	1.12 (0.62, 2.02)	0.628
Facility type (1 = PNFP)	1.40 (0.70, 2.78)	0.211	1.52 (0.67, 3.42)	0.185
Pediatric HIV support	0.99 (0.67, 1.46)	0.927	0.90 (0.58, 1.41)	0.552
CQI experience	0.73 (0.51, 1.04)	0.023	0.75 (0.50, 1.12)	0.066
DEA stationed	1.13 (0.95, 1.34)	0.063	1.17 (0.99, 1.40)	0.018
EIR (ref: very low/no prevalence)				
Low	1.09 (0.56, 2.15)	0.731	1.00 (0.45, 2.22)	0.992
Medium – High	1.02 (0.51, 2.02)	0.941	1.04 (0.45, 2.38)	0.906
Very high	1.03 (0.59, 1.78)	0.898	0.95 (0.48, 1.87)	0.836
Ideal clinical staff ratio quartile (ref: lowest)				
2^nd^	1.12 (0.76, 1.65)	0.446	0.97 (0.63, 1.49)	0.859
3^rd^	0.62 (0.35, 1.09)	0.030	0.56 (0.30, 1.05)	0.017
Highest	1.30 (0.90, 1.88)	0.068	1.19 (0.75, 1.90)	0.322
Clinically active staff quartile (ref: lowest)				
2^nd^	1.08 (0.89, 1.30)	0.309	1.03 (0.82, 1.29)	0.717
3^rd^	1.20 (0.94, 1.53)	0.053	1.13 (0.86, 1.48)	0.257
Highest	1.32 (0.93, 1.87)	0.044	1.26 (0.85, 1.88)	0.132
Lab staff quartile (ref: lowest)				
2^nd^	0.99 (0.71, 1.37)	0.930	1.01 (0.70, 1.47)	0.928
3^rd^	1.09 (0.77, 1.53)	0.536	1.05 (0.49, 1.57)	0.752
Highest	1.05 (0.67, 1.65)	0.793	0.87 (0.49, 1.55)	0.534

p<0.01.

**Table 5:** Adjusted relative risk across time periods and arms for appropriate antimalarial treatment.

	Proportion of patients prescribed an appropriate anti-malarial among those with any antimalarial prescription		Proportion of patients that received an appropriate antimalarial among those with an antimalarial appropriate prescription and data about drug availability		Estimated proportion of patients that received an appropriate antimalarial among those with any antimalarial prescription	
Sub groups	Under 5	5 and above	Under 5	5 and above	Under 5	5 and above
**Part 1: Percentage**						
**Arm A**						
Time 0	16,947 (91%)	27,004 (82%)	4,850 (58%)	7,316 (56%)	9,467 (51%)	14,040 (42%)
Time 1	36,126 (97%)	57,822 (95%)	23,565 (81%)	37,918 (79%)	28,434 (81%)	44,192 (73%)
**% Change**	**6%**	**13%**	**23%**	**23%**	**26%**	**30%**
**Arm B**						
Time 0	41,644 (95%)	54,844 (87%)	18,147 (66%)	29,417 (72%)	25,002 (57%)	35,827 (57%)
Time 1	39,483 (96%)	62,591 (87%)	23,469 (69%)	43,625 (75%)	27,368 (66%)	46,520 (65%)
**% Change**	**1%**	**0%**	**2%**	**3%**	**9%**	**8%**
**Part II: Regression**	**aRR (CI)**	**p-value**	**aRR (CI)**	**p-value**	**aRR (CI)**	**p-value**
**Arm A:** T1 – T0	1.10 (0.99, 1.22)	0.016	1.51* (1.05, 2.15)	0.003	1.50* (1.04, 2.17)	0.004
**Arm B:** T1 – T0	0.99 (0.95, 1.02)	0.332	1.09 (0.89, 1.34)	0.279	1.09 (0.87, 1.36)	0.315
**Arm A vs. Arm B:**						
T1-T0 **(aRRR)**	1.11 (0.99, 1.25)	0.017	1.38 (0.94, 2.04)	0.033	1.38 (0.89, 2.13)	0.061
**Covariates**						
Age (1 = Under 5)	1.08* (1.03, 1.13)	0.000	0.98 (0.93, 1.03)	0.287	1.06 (0.99, 1.13)	0.023
Facility level (1 = Hospital)	0.94 (0.85, 1.04)	0.130	1.19 (0.88, 1.62)	0.142	1.05 (0.70, 1.56)	0.760
Facility type (1 = PNFP)	1.02 (0.87, 1.19)	0.780	1.18 (0.83, 1.67)	0.218	1.23 (0.72, 2.10)	0.309
Pediatric HIV support	1.04 (0.97, 1.12)	0.120	1.29 (0.96, 1.74)	0.027	1.37 (0.99, 1.89)	0.013
CQI experience	1.02 (0.95, 1.11)	0.420	0.98 (0.73, 1.32)	0.851	1.07 (0.76, 1.51)	0.599
DEA stationed	1.02 (0.98, 1.06)	0.192	0.88 (0.75, 1.04)	0.044	1.05 (0.82, 1.35)	0.618
EIR (ref: very low/no prevalence)						
Low	1.02 (0.84, 1.23)	0.789	0.61* (0.38, 0.99)	0.009	0.75 (0.39, 1.45)	0.263
Medium – High	0.82* (0.67, 0.99)	0.007	0.90 (0.64, 1.26)	0.420	0.78 (0.51, 1.20)	0.137
Very high	0.97 (0.81, 1.15)	0.592	0.77 (0.53, 1.12)	0.075	0.88 (0.52, 1.49)	0.537
Ideal clinical staff ratio quartile (ref: lowest)						
2^nd^	1.08 (0.99, 1.17)	0.025	1.11 (0.75, 1.64)	0.510	1.24 (0.82, 1.87)	0.188
3^rd^	1.07 (0.99, 1.15)	0.034	1.43* (1.01, 2.03)	0.008	1.62* (1.16, 2.26)	<0.001
Highest	1.12* (1.01, 1.24)	0.004	1.19 (0.80, 1.77)	0.255	1.47 (0.95, 2.26)	0.023
Clinically active staff quartile (ref: lowest)						
2^nd^	1.01 (0.97, 1.05)	0.535	0.91 (0.78, 1.07)	0.133	0.96 (0.79, 1.15)	0.535
3^rd^	1.02 (0.96, 1.07)	0.449	0.96 (0.78, 1.18)	0.590	0.95 (0.74, 1.22)	0.584
Highest	1.04 (0.98, 1.10)	0.134	0.79 (0.59, 1.07)	0.047	0.89 (0.65, 1.20)	0.309

p<0.*0*1.

**Table 6:** Adjusted relative risk across time periods and arms of prescribing based on diagnostic malaria test result.

	Proportion of patients with a negative diagnostic test result for malaria who were prescribed an antimalarial		Proportion of patients with a positive diagnostic test result for malaria who were prescribed an antibiotic	
Sub groups	Under 5	5 and above	Under 5	5 and above
**Part 1: Percentage**				
**Arm A**				
Time 0	1,884 (56%)	3,528 (42%)	2,830 (48%)	2,271 (41%)
Time 1	4,017 (37%)	8,374 (27%)	7,714 (50%)	6,688 (41%)
**% Change**	**−19%**	**−15%**	**2%**	**0%**
**Arm B**				
Time 0	5,424 (65%)	7,519 (47%)	5,613 (52%)	4,149 (41%)
Time 1	6,334 (60%)	9,351 (44%)	5,601 (54%)	4,293 (44%)
**% Change**	**−5%**	**−3%**	**2%**	**2%**
**Part II: Regression**	**aRR (CI)**	**p-value**	**aRR (CI)**	**p-value**
**Arm A:** T1 – T0	0.67* (0.46, 0.97)	0.006	1.04 (0.88, 1.21)	0.566
**Arm B:** T1 – T0	0.96 (0.84, 1.10)	0.400	1.06 (0.96, 1.17)	0.124
**Arm A vs. Arm B:**				
T1-T0 **(aRRR)**	0.70 (0.48, 1.00)	0.011	0.98 (0.81, 1.18)	0.747
**Covariates**				
Age (1 = Under 5)	1.39* (1.25, 1.55)	<0.001	1.25* (1.14, 1.36)	<0.001
Facility level (1 = Hospital)	1.25 (0.77, 2.03)	0.242	1.15 (0.82, 1.63)	0.284
Facility type (1 = PNFP)	1.15 (0.59, 2.23)	0.596	0.90 (0.69, 1.18)	0.323
Pediatric HIV support	0.90 (0.62, 1.31)	0.465	0.90 (0.67, 1.21)	0.358
CQI experience	1.29 (0.76, 2.19)	0.224	1.23 (0.90, 1.69)	0.092
DEA stationed	0.95 (0.83, 1.09)	0.381	1.02 (0.90, 1.15)	0.731
EIR (ref: very low/no prevalence)				
Low	1.66 (0.64, 4.31)	0.174	0.53 (0.21, 1.30)	0.067
Medium – High	1.29 (0.69, 2.41)	0.298	1.28 (0.78, 2.09)	0.206
Very high	1.07 (0.47, 2.45)	0.824	0.88 (0.54, 1.45)	0.519
Ideal clinical staff ratio quartile (ref: lowest)				
2^nd^	1.09 (0.67, 1.76)	0.654	1.05 (0.74, 1.48)	0.711
3^rd^	0.86 (0.41, 1.80)	0.598	1.10 (0.72, 1.69)	0.565
Highest	1.10 (0.64, 1.89)	0.657	0.92 (0.63, 1.35)	0.581
Clinically active staff quartile (ref: lowest)				
2^nd^	0.94 (0.74, 1.20)	0.520	1.03 (0.88, 1.22)	0.613
3^rd^	0.95 (0.74, 1.22)	0.587	1.04 (0.88, 1.24)	0.527
Highest	1.10 (0.86, 1.30)	0.319	1.28* (1.03, 1.50)	0.003

p<0.01.

### Outcomes and Estimation

#### Parasitological diagnosis

The results for the proportion of patients with suspected malaria for whom a diagnostic test result for malaria was recorded are reported in Table 4. The indicator was reported in two steps as the proportion of patients with suspected malaria: a) for whom a diagnostic test for malaria was ordered, and b) for whom a diagnostic test result for malaria was recorded. The steps distinguish the clinicians' practices from the laboratory capacity and laboratory personnel practices. Between Time 0 and Time 1, the proportion of patients with suspected malaria for whom a diagnostic test for malaria was ordered increased by 28% in arm A compared to a 1% decrease in arm B. The proportion of patients with suspected malaria for whom a malaria diagnostic test result was recorded increased by 25% in arm A compared to a 3% decrease in arm B. The 28% difference between the changes in arm A and arm B was attributable to OSS (adjusted ratio of relative risks (aRRR) = 1.28, 99%CI: 0.93, 1.78). Patients under five years with suspected malaria were 14% (aRR = 1.14 99% CI: 1.00–1.30) more likely to have a diagnostic test result for malaria recorded than older patients.

#### Appropriate antimalarial treatment

Table 5 shows results for (a) proportion of patients prescribed an appropriate antimalarial among those with any antimalarial prescription, (b) proportion of patients that received an appropriate antimalarial among those with an appropriate antimalarial prescription and data about drug availability and (c) estimated proportion of patients that received an appropriate antimalarial among those prescribed any antimalarial treatment. In arm A, the proportion of patients prescribed an appropriate antimalarial increased by 10% and the proportion of patients who received an appropriate antimalarial among those with an appropriate antimalarial prescription and data on drug availability increased by 51% between Time 0 and Time 1. At the same time, the estimated proportion of patients who received an appropriate antimalarial among those prescribed any antimalarial increased by 50%. The increases were statistically significant for indicators (b) and (c) in arm A. The indicators were relatively stable in arm B. The difference between arms attributed to OSS was 11% (aRRR = 1.11; 99%CI = 0.99, 1.25), 38% (aRRR = 1.38; 99%CI =  0.94, 2.04), and 38% (aRRR = 1.38; 99%CI = 0.89, 2.13), respectively.

#### Antimalarial treatment among patients with a negative diagnostic test result for malaria

Table 6 shows that the proportion of patients with a negative diagnostic test result for malaria prescribed an antimalarial decreased significantly by 33% in arm A and decreased by 4% in arm B, with a 30% difference attributed to OSS (aRRR = 0.70, 99%CI: 0.48, 1.00). Patients under five years with a negative diagnostic test result for malaria were 39% more likely to be prescribed an antimalarial than older patients (aRR = 1.39, 99%CI: 1.25, 1.55).

#### Antibiotic treatment among patients with a positive diagnostic test result for malaria

Table 6 shows that, the proportion of patients with a positive diagnostic test result for malaria prescribed an antibiotic (with or without an accompanying antimalarial) did not change in either arm. Patients under five years with a positive diagnostic test result for malaria were however, 25% (aRR = 1.25, 99%CI: 1.14, 1.36) more likely to be prescribed an antibiotic than older patients.

### Sensitivity Analysis

In sensitivity analyses, the results were robust in variance estimates with bootstrapping and estimates that omitted outliers and influential observations with one exception. For the estimated proportion of patients that received an appropriate antimalarial, the standard error of the coefficient increased and the p-value dropped to 0.05. For this same indicator, the results did not change when the women who were prescribed SP monotherapy were omitted from the analysis. In estimates without the variable for DEA stationed at the site, the coefficients for the interventions generally showed larger effect sizes and smaller standard errors.

### Measure of Dispersion

**Figure 3:**
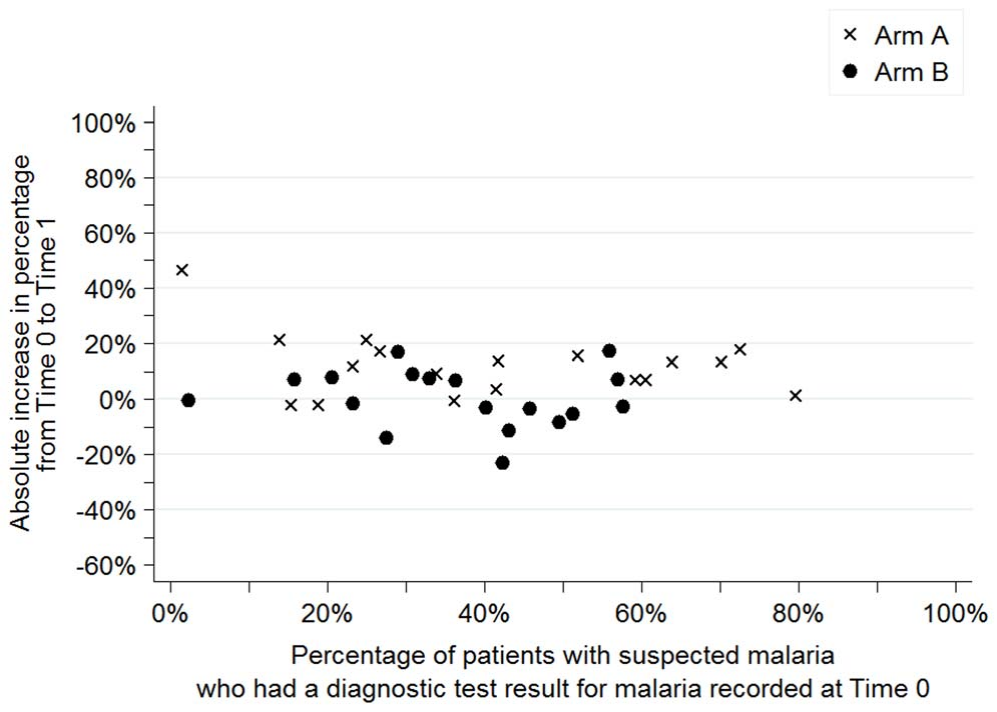
Percentage of patients with suspected malaria who had a diagnostic test result for malaria recorded, dispersion by facility and arm.

**Figure 4:**
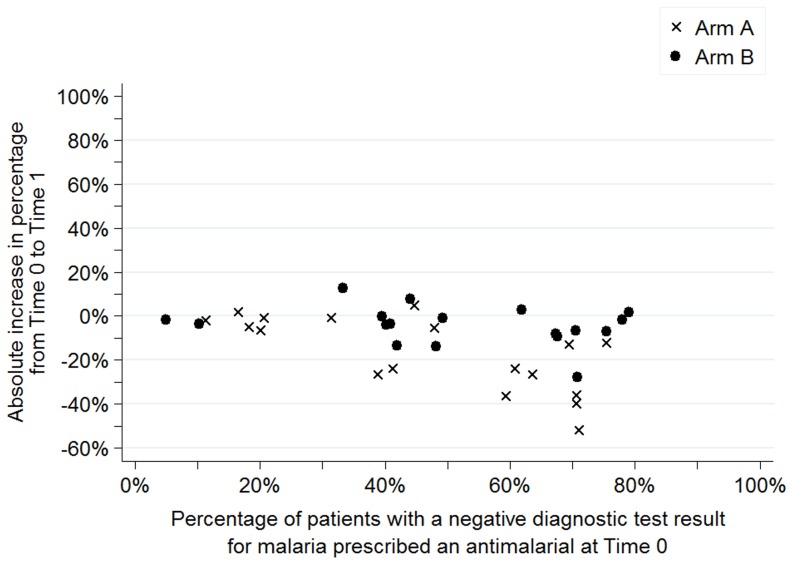
Percentage of patients with a negative diagnostic test result for malaria who were prescribed an antimalarial, dispersion by facility and arm.

**Figure 5:**
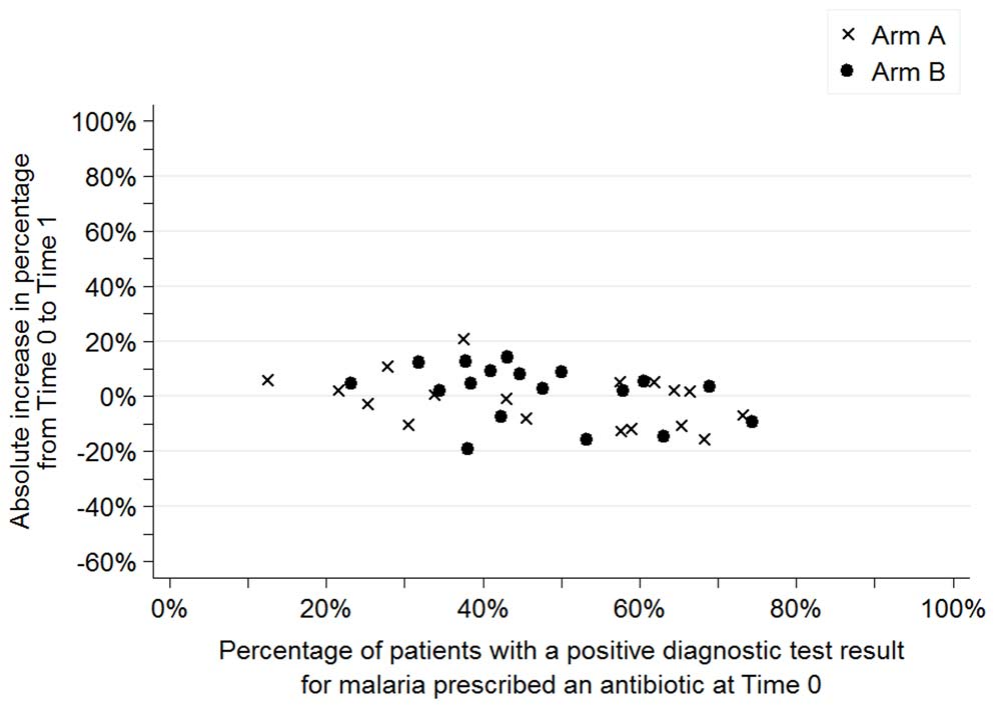
Percentage of patients with a positive diagnostic test result for malaria who were prescribed an antibiotic, dispersion by facility and arm.

**Figure 6:**
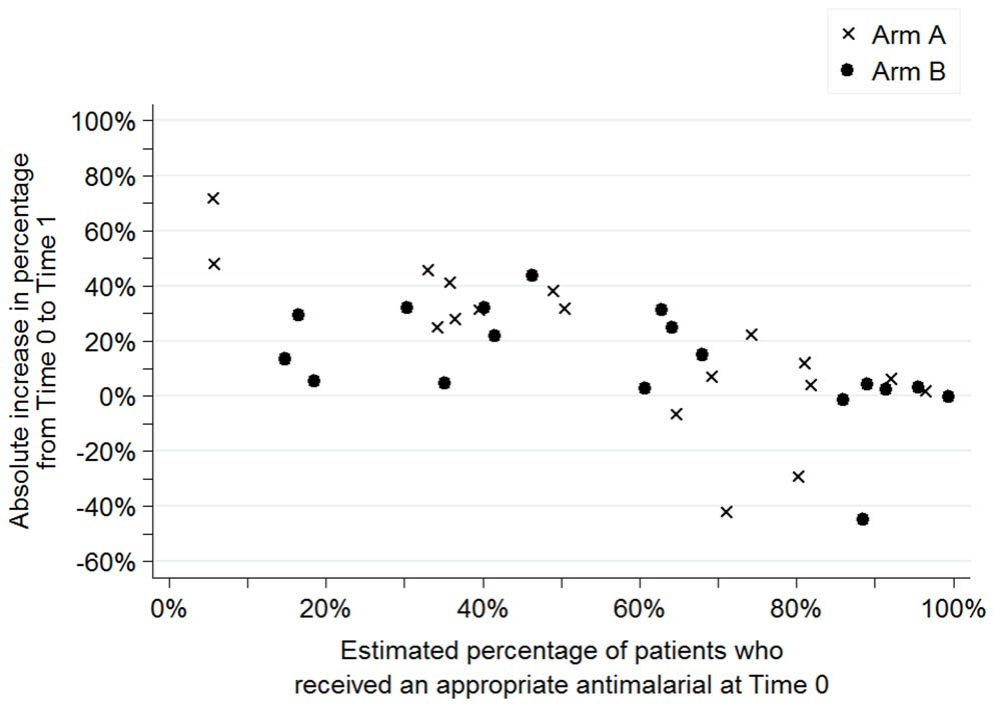
Estimated percentage of patients who received an appropriate antimalarial, dispersion by facility and arm.

Figures 3 to 6 show the baseline values and improvement for all sites for each of the four indicators. Values at Time 0 are on the horizontal axis and the absolute change in percentage between Time 0 and Time 1 are on the vertical axis. Arm A sites are noted by an “x” and arm B sites are noted by a dot.

As shown in Figure 3, Time 0 values for the percentage of patients with suspected malaria for whom a test result was recorded ranged from 2% to 80% in arm A and from 4% to 58% in arm B with the majority (12 in arm A and 13 in arm B) of the sites recording results for less than 50% of the patients with suspected malaria. Regardless of performance at Time 0, sites in arm A had greater improvements than sites arm B as shown by the “x”s above the dots for most baseline values.

Figure 4 shows that the range of values at Time 0 for the estimated percentage of malaria cases that received an appropriate antimalarial was broad in both arms. Again, sites in arm A had greater improvements than arm B, with 10 sites showing improvements of more than 20% in arm A compared to seven sites in arm B.

Figure 5 shows the percentage of patients with a negative diagnostic test result for malaria prescribed an antimalarial. For this indicator, the distribution of sites was comparable across arms at baseline. Regardless of performance at Time 0, sites in arm A had greater improvements than sites in arm B as shown by the “x's” below the dots for most baseline values.

Finally, Figure 6 shows that the percentage of patients with a positive diagnostic test result for malaria prescribed an antibiotic at Time 0 clustered between 20% and 80% in both arms. Changes over time were relatively small with no clear difference across arms.

### Exploratory Analysis

Results in Table 7 revealed that the proportion of patients with a negative diagnostic test result for malaria who were subsequently diagnosed with malaria was relatively high among children under five years (41% in arm A and 52% in arm B) and adults (31% in arm A and 38% in arm B) at Time 0. This proportion reduced by 35% in arm A but remained the same in arm B at Time 1. Compared to the results in Table 6 for patients with a negative diagnostic test result for malaria, the number with a malaria diagnosis is always less than the number prescribed an antimalarial. Children under five years with a negative diagnostic test result for malaria were 34% (aRR = 1.34, 99%CI: 1.23, 1.45) more likely to be diagnosed with malaria than older patients.

**Table 7:** Exploratory analysis.

	Proportion of patients with a malaria diagnosis among those with a negative diagnostic test result for malaria		Proportion of patients with a malaria diagnosis among those with a positive diagnostic test result for malaria		Proportion of patients with a positive diagnostic test result for malaria among those with a malaria diagnosis	
Sub groups	Under 5	5 and above	Under 5	5 and above	Under 5	5 and above
**Part I: Percentage**						
**Arm A**						
Time 0	1,390 (41%)	2,571 (31%)	4,865 (82%)	4,515 (81%)	4,865 (30%)	4,515 (15%)
Time 1	3,154 (29%)	6,627 (22%)	14,503 (94%)	15,209 (92%)	14,503 (42%)	15,209 (27%)
**% Change**	**−12%**	**−9%**	**11%**	**11%**	**13%**	**12%**
**Arm B**						
Time 0	4,317 (52%)	6,080 (38%)	8,605 (79%)	8,202 (82%)	8,605 (22%)	8,202 (14%)
Time 1	5,545 (53%)	8,778 (41%)	8,837 (85%)	8,726 (89%)	8,837 (23%)	8,726 (12%)
**% Change**	**1%**	**3%**	**5%**	**7%**	**1%**	**−2%**
**Part II: Regression**	**aRR (CI)**	**p-value**	**aRR (CI)**	**p-value**	**aRR (CI)**	**p-value**
**Arm A:** T1 – T0	0.65 (0.40, 1.05)	0.020	1.03 (0.93, 1.13)	0.496	1.34 (0.92, 1.95)	0.048
**Arm B:** T1 – T0	1.01 (0.90, 1.13)	0.860	1.01 (0.94, 1.09)	0.651	0.88 (0.63, 1.22)	0.305
**Arm A vs. Arm B:**						
T1-T0 **(aRRR)**	0.65 (0.40, 1.05)	0.021	1.01 (0.90, 1.14)	0.758	1.52 (0.96, 2.42)	0.019
**Covariates**						
Age (1 = Under 5)	1.34* (1.23, 1.45)	<0.001	0.99 (0.96, 1.02)	0.386	1.50* (1.23, 1.84)	<0.001
Facility level (1 = Hospital)	1.20 (0.76, 1.90)	0.293	1.01 (0.92, 1.10)	0.814	1.13 (0.39, 3.29)	0.776
Facility type (1 = PNFP)	1.01 (0.38, 2.67)	0.978	1.02 (0.93, 1.13)	0.525	2.40 (0.64, 9.05)	0.090
Pediatric HIV support	0.76 (0.48, 1.21)	0.129	0.90* (0.82, 0.99)	0.006	0.85 (0.47, 1.54)	0.487
CQI experience	1.57 (0.84, 2.92)	0.064	0.99 (0.92, 1.06)	0.635	0.67 (0.39, 1.16)	0.062
DEA stationed	1.09 (0.92, 1.29)	0.199	1.14* (1.03, 1.25)	0.001	1.23 (0.97, 1.55)	0.025
EIR (ref: very low/no prevalence)						
Low	1.20 (0.40, 3.59)	0.668	1.12 (0.90, 1.40)	0.170	2.52 (0.35, 18.15)	0.228
Medium – High	1.05 (0.40, 2.71)	0.902	1.06 (0.86, 1.31)	0.444	4.30 (0.62, 29.66)	0.052
Very high	0.95 (0.39, 2.33)	0.892	1.08 (0.89, 1.33)	0.299	4.01 (0.60, 26.96)	0.060
Ideal clinical staff ratio quartile (ref: lowest)						
2^nd^	1.15 (0.69, 1.93)	0.468	1.05 (0.97, 1.13)	0.100	1.04 (0.57, 1.90)	0.855
3^rd^	1.01 (0.48, 2.11)	0.973	1.04 (0.96, 1.14)	0.213	0.42* (0.18, 0.99)	0.009
Highest	1.12 (0.43, 2.91)	0.757	1.05 (0.98, 1.12)	0.086	1.22 (0.62, 2.39)	0.449
Clinically active staff quartile (ref: lowest)						
2^nd^	0.85 (0.67, 1.08)	0.077	1.02 (0.96, 1.07)	0.454	0.98 (0.69, 1.40)	0.892
3^rd^	0.90 (0.71, 1.14)	0.245	1.01 (0.97, 1.06)	0.496	1.05 (0.73, 1.51)	0.735
Highest	1.01 (0.77, 1.34)	0.900	0.99 (0.93, 1.04)	0.526	1.23 (0.74, 2.03)	0.289
Lab staff quartile (ref: lowest)						
2^nd^	1.44 (0.76, 2.73)	0.143	0.94 (0.83, 1.07)	0.203	0.79 (0.50, 1.25)	0.186
3^rd^	1.36 (0.71, 2.63)	0.226	0.99 (0.90, 1.09)	0.740	0.80 (0.46, 1.39)	0.303
Highest	1.28 (0.30, 5.57)	0.660	0.98 (0.85, 1.14)	0.725	0.50 (0.16, 1.55)	0.114

p<0.01.

The proportion of patients with a malaria diagnosis among those with a positive diagnostic test result for malaria was high in both arms at Times 0 and 1 and did not increase significantly in either arm.

Interestingly, the proportion of patients with a positive diagnostic test result for malaria among those with a malaria diagnosis was low in both arms at Time 0. The proportion increased by 34% in arm A in Time 1, while it decreased by 12% in arm B. Children under five years with a malaria diagnosis were 50% (aRR = 1.50, 99%CI: 1.23, 1.84) more likely to have a positive diagnostic test result for malaria than older patients.

## Discussion

### Interpretation

This article focused on four malaria case management indicators out of 23 facility performance indicators analyzed for IDCAP. The pre/post analysis showed that IMID was not associated with large or statistically significant improvements in those indicators. A combination of both IMID and OSS was however, associated with statistically significant improvements in two indicators: estimated proportion of patients who received an appropriate antimalarial among those prescribed any antimalarial treatment and proportion of patients with a negative diagnostic test result for malaria prescribed an antimalarial.

These results for patients with a negative diagnostic test result for malaria were encouraging after several previous studies showed that increased use of microscopy did not guarantee reduced prescription of antimalarials among patients with a negative diagnostic test result for malaria [8], [19]–[23]. The IDCAP results suggest that clinicians trusted the laboratory test results during the OSS intervention that included team-based training, as well as training and mentoring for laboratory professionals. In addition, reducing prescriptions of antimalarials among patients with a negative diagnostic test result for malaria may leave a larger supply of drugs available to patients with a positive diagnostic test result for malaria.

The effect of OSS measured by the randomized trial was not statistically significant at the 1% level for any of the four indicators. The effects sizes were large however, for three of the four indicators: 28% increase in the proportion of patients with suspected malaria for whom a diagnostic test result for malaria was recorded, 38% increase in the estimated proportion of patients that received an appropriate antimalarial, and 30% decrease in the proportion of patients with a negative test result for malaria prescribed an antimalarial treatment.

The IDCAP results compared favorably to the earlier JUMP results [12]. IDCAP's pre/post analysis of IMID and OSS showed a 25% increase in the proportion of patients with suspected malaria for whom a diagnostic test for malaria was recorded, compared to JUMP's 16 and 19% increases in the percentage of children under five years and older patients, respectively, referred for microscopy. IDCAP showed a 49% increase in the estimated proportion of patients who received an appropriate antimalarial, compared to JUMP's increases of three and one percent, respectively, which were not statistically significant. IDCAP showed a 33% decrease in the proportion of patients with a negative diagnostic test result for malaria prescribed an antimalarial, compared to JUMP's decreases of 28% and 23%, respectively. The JUMP tests were based on a 5% level of significance, and two of these IDCAP results were significant at that level. Finally, nine months of follow-up data in arm A were analyzed in IDCAP compared to four months in JUMP. UMSP later showed continued improvements with continued data collection [9].

There were important differences between the IDCAP interventions and JUMP besides the scope. Only two MLP from each site attended IMID, whereas a multi-disciplinary team of clinicians, laboratory professionals, and records staff attended JUMP. The multidisciplinary OSS activities were structured over the course of two days per month, whereas the JUMP follow-up visits focused on data collection and were briefer. To the extent that IMID affected the performance of the two MLP, the effect may not have been reflected in overall facility performance. OSS would however, reflect the effects of multi-disciplinary team training on overall facility performance. Future analyses will compare the performance of the two MLP who attended IMID with the other clinicians.

IDCAP was implemented at the same time as the data collection system that measured its effects, which could be considered an intervention. For the JUMP evaluation, it was not possible to distinguish the effects of the training program from UMSP's data collection [9], [24]. IDCAP controlled for the effects of the data collection system, which was based on the UMSP platform, and offered a model for addressing the simultaneous effects of capacity-building and data collection. The IDCAP data management system was introduced at all the sites by November 2009, and the DEA variable measured the effect of the full-time DEA at the sites beginning in March 2010. The DEA were not associated with significant changes in the malaria case management indicators at the 1% level of significance. If we relax the standard of evidence to a 5% level of significance, the DEA were associated with a 17% increase in the proportion of patients with suspected malaria for whom a diagnostic test result for malaria was recorded.

Neither IMID nor a combination of IMID and OSS affected antibiotic prescription among patients with a positive diagnostic test result for malaria. The IDCAP interventions admittedly focused more on case management of patients with a negative diagnostic test result for malaria than on limiting antibiotic use among patients with a positive diagnostic test result for malaria. Antibiotic use appeared high however; for both IDCAP arms and time periods combined, 51% of children under five years with a positive diagnostic test result for malaria were prescribed an antibiotic. In comparison, Batwala et al., recently reported that 26% of children with a positive rapid diagnostic test result and 18% with positive laboratory test results were prescribed antibiotic treatment in Uganda [25]. In Means et al.'s (unpublished manuscript) investigation of antibiotic use among patients with a positive diagnostic test result for malaria, they conducted separate analyses for patients with a clinical indication for antibiotics and patients without one. This distinction could be the basis for a revised indicator for antibiotic use among patients with positive diagnostic test results for malaria.

From the results of exploratory analysis, a high number of patients with a negative diagnostic test result for malaria, especially children under five, were diagnosed with malaria. Even though the numbers reduced by more than 30% after OSS, convincing the clinicians to believe in laboratory results was an uphill task. Also, close to 20% of patients with a positive test result for malaria were not diagnosed with malaria. The results improved following OSS and the presence of the DEA significantly contributed to these improvements. The numbers who were diagnosed with malaria without any laboratory confirmation remained very high.

### Limitations

In the definition of a patient with suspected malaria, we considered patients with any of the following four criteria: fever, malaria test ordered, malaria diagnosis, and any malaria treatment prescribed. This definition is slightly different from the current definitions being used by the Uganda Ministry of Health and could have overestimated the total number of patients with suspected malaria. The appropriate treatment for malaria variable used was based on the Uganda national guidelines, but did not measure appropriate dose or duration of treatment.

### Generalizability

IDCAP's eligibility criteria focused on HC IV that were not currently participating in on-going national CQI programs for HIV prevention and care to isolate the effect of OSS. Although the criteria would restrict generalizability of the results to only a handful of other facilities in Uganda, the confounding effect of an HIV program may be less relevant for malaria case management. The results may generalize to other primary care facilities that serve populations at risk for malaria in Africa.

The statistical tests addressed whether or not the results would generalize to other primary care facilities. The IDCAP tests were based on a 1% level of significance to adjust for multiple comparisons, because we tested the effects of the integrated intervention on 23 facility-performance indicators. Only two indicators met this standard of evidence in the pre/post analysis.

Replication also provides evidence on whether or not the results would generalize to other primary care facilities. Both JUMP and IDCAP showed training and on-site support significantly improved case management for malaria, albeit at the 5% level of significance.

## Conclusions

The combination of IMID and OSS was associated with statistically significant improvements in case management of malaria. A series of papers will provide results on other performance indicators and cost-effectiveness.

## Supporting Information

Protocol S1 **IDCAP research protocol.**
(DOC)

Checklist S1 **CONSORT checklist for documentation of the article content.**
(DOCX)

## References

[1] World Health Organization (2012) World Malaria Report 2012. Geneva.

[2] Government of Uganda (2004), *Health Sector Strategic Plan II, 2004/05-2009/10* Ministry of Health. Available: http://aidsalliance.3cdn.net/e9266246309cee49e2_qbm6bt9wn.pdf. Accessed 2013 Sep 14.

[3] Government of Uganda (2010) *Uganda National Household Survey 2009/10*. Uganda Bureau of Statistics.

[4] Government of Uganda (2011) Uganda National Malaria Control Strategic Plan 2010/11-2014/15. Ministry of Health.

[5] Government of Uganda (2011) *Annual Health Sector Performance Report 2010/2011*. Ministry of Health.

[6] World Health Organization (2006) Guidelines for the treatment of malaria: second edition. Geneva.

[7] Ndyomugyenyi R, Magnussen P, Clarke S (2007) Malaria treatment-seeking behaviour and drug prescription practices in an area of low transmission in Uganda: implications for prevention and control. Trans R Soc Trop Med Hyg 101: 209–215 S0035-9203(06)00202-1 [pii]; 10.1016/j.trstmh.2006.06.004 [doi] 16950487

[8] Hamer DH, Ndhlovu M, Zurovac D, Fox M, Yeboah-Antwi K, et al (2007) Improved diagnostic testing and malaria treatment practices in Zambia. JAMA 297: 2227–2231 297/20/2227 [pii]; 10.1001/jama.297.20.2227 [doi] 17519412 PMC2674546

[9] Sserwanga A, Harris JC, Kigozi R, Menon M, Bukirwa H, et al (2011) Improved malaria case management through the implementation of a health facility-based sentinel site surveillance system in Uganda. PLoS One 6: e16316 10.1371/journal.pone.0016316 [doi] 21283815 PMC3023768

[10] Osterholt DM, Rowe AK, Hamel MJ, Flanders WD, Mkandala C, et al (2006) Predictors of treatment error for children with uncomplicated malaria seen as outpatients in Blantyre district, Malawi. Trop Med Int Health 11: 1147–1156 TMI1666 [pii]; 10.1111/j.1365-3156.2006.01666.x [doi] 16903878

[11] Opoka RO, Xia Z, Bangirana P, John CC (2008) Inpatient mortality in children with clinically diagnosed malaria as compared with microscopically confirmed malaria. Pediatr Infect Dis J 27: 319–324 10.1097/INF.0b013e31815d74dd [doi] 18316995 PMC2607243

[12] Ssekabira U, Bukirwa H, Hopkins H, Namagembe A, Weaver MR, et al.. (2008) Improved malaria case management after integrated team-based training of health care workers in Uganda. Am J Trop Med Hyg 79: 826–833. 79/6/826 [pii]. 19052287

[13] Miceli A, Sebuyira LM, Crozier I, Cooke M, Naikoba S, et al (2012) Advances in clinical education: a model for infectious disease training for mid-level practitioners in Uganda. Int J Infect Dis 16: e708–e713 S1201-9712(12)01208-8 [pii]; 10.1016/j.ijid.2012.07.003 [doi] 22906682

[14] Weaver MR, Crozier I, Eleku S, Makanga G, Mpanga SL, et al (2012) Capacity-building and clinical competence in infectious disease in Uganda: a mixed-design study with pre/post and cluster-randomized trial components. PLoS One 7: e51319 10.1371/journal.pone.0051319 [doi];PONE-D-12-22600 [pii] 23272097 PMC3522731

[15] Naikoba S, Colebunders R, van Geertruyden JP, Willis SK, Kinoti NS, et al (2013) Design of a cluster randomized trial assessing integrated infectious diseases training and on-site support for midlevel practitioners in Uganda. Journal of Clinical Care Pathways 0: 1–8.

[16] Government of Uganda (2005) National Policy on Malaria Treatment 2005. Ministry of Health.

[17] Lumley T, Kronmal R, Ma S. Relative Risk Regression in Medical Research: Models, Contrasts, Estimators, and Algorithms. University of Washington Biostatistics Working Paper #293. This working paper site is hosted by The Berkeley Electronic Press (bepress). Available: http://www.bepress.com/uwbiostat/paper293. Accessed 2013 Sep 4.

[18] Yeka A, Gasasira A, Mpimbaza A, Achan J, Nankabirwa J, et al (2012) Malaria in Uganda: challenges to control on the long road to elimination: I. Epidemiology and current control efforts. Acta Trop 121: 184–195 S0001-706X(11)00061-1 [pii]; 10.1016/j.actatropica.2011.03.004 [doi] 21420377 PMC3156969

[19] Yeka A, Banek K, Bakyaita N, Staedke SG, Kamya MR, et al (2005) Artemisinin versus nonartemisinin combination therapy for uncomplicated malaria: randomized clinical trials from four sites in Uganda. PLoS Med 2: e190 05-PLME-RA-0041R3 [pii]; 10.1371/journal.pmed.0020190 [doi] 16033307 PMC1181876

[20] Reyburn H, Mbakilwa H, Mwangi R, Mwerinde O, Olomi R, et al (2007) Rapid diagnostic tests compared with malaria microscopy for guiding outpatient treatment of febrile illness in Tanzania: randomised trial. BMJ 334: 403 bmj.39073.496829.AE [pii]; 10.1136/bmj.39073.496829.AE [doi] 17259188 PMC1804187

[21] Reyburn H, Ruanda J, Mwerinde O, Drakeley C (2006) The contribution of microscopy to targeting antimalarial treatment in a low transmission area of Tanzania. Malar J 5: 4 1475–2875-5-4 [pii]; 10.1186/1475-2875-5-4 [doi] 16423307 PMC1360087

[22] Zurovac D, Midia B, Ochola SA, English M, Snow RW (2006) Microscopy and outpatient malaria case management among older children and adults in Kenya. Trop Med Int Health 11: 432–440 TMI1587 [pii]; 10.1111/j.1365-3156.2006.01587.x [doi] 16553926

[23] Barat L, Chipipa J, Kolczak M, Sukwa T (1999) Does the availability of blood slide microscopy for malaria at health centers improve the management of persons with fever in Zambia? Am J Trop Med Hyg 60: 1024–1030. 10403337 10.4269/ajtmh.1999.60.1024

[24] Namagembe A, Ssekabira U, Weaver MR, Blum N, Burnett S, et al (2012) Improved clinical and laboratory skills after team-based, malaria case management training of health care professionals in Uganda. Malar J 11: 44 1475-2875-11-44 [pii]; 10.1186/1475-2875-11-44 [doi] 22330281 PMC3342908

[25] Batwala V, Magnussen P, Nuwaha F (2011) Antibiotic use among patients with febrile illness in a low malaria endemicity setting in Uganda. Malar J 10: 377 1475-2875-10-377 [pii]; 10.1186/1475-2875-10-377 [doi] 22183039 PMC3258227

